# Content and discontent: a qualitative exploration of obstacles to elearning engagement in medical students

**DOI:** 10.1186/s12909-016-0710-5

**Published:** 2016-07-22

**Authors:** Helen J. Reid, Clare Thomson, Kieran J. McGlade

**Affiliations:** School of Medicine, Dentistry and Biomedical Sciences, Queen’s University Belfast, University Road, Belfast, BT7 1NN UK; Department of General Practice, Dunluce Health Centre, 1 Dunluce Avenue, Belfast, BT9 7HR UK

**Keywords:** Elearning, Health professions education, Undergraduate, Achievement emotions, Obstacles

## Abstract

**Background:**

Elearning is ubiquitous in healthcare professions education. Its equivalence to ‘traditional’ educational delivery methods is well established. There is a research imperative to clarify *when* and *how* to use elearning most effectively to mitigate the potential of it becoming merely a ‘disruptive technology.’ Research has begun to broadly identify challenges encountered by elearning users. In this study, we explore in depth the perceived obstacles to elearning engagement amongst medical students. Sensitising concepts of achievement emotions and the cognitive demands of multi-tasking highlight why students’ deeply emotional responses to elearning may be so important in their learning.

**Methods:**

This study used focus groups as a data collection tool. A purposeful sample of 31 participated. Iterative data gathering and analysis phases employed a constant comparative approach to generate themes firmly grounded in participant experience.

**Results:**

Key themes that emerged from the data included a sense of injustice, passivity and a feeling of being ‘lost at sea’. The actual content of the elearning resource provided important context.

**Conclusions:**

The identified themes have strong emotional foundations. These responses, interpreted through the lens of achievement emotions, have not previously been described. Appreciation of their importance is of benefit to educators involved in curriculum development or delivery.

**Electronic supplementary material:**

The online version of this article (doi:10.1186/s12909-016-0710-5) contains supplementary material, which is available to authorized users.

## Background

Elearning, computer based learning, technology-enhanced learning…. irrespective of fashionable terminology, technology is an intrinsic feature of healthcare professions education [1]. Ellaway’s simple definition of elearning as, *“a loosely defined amalgam of information communication technologies (ICTs) used in education, usually but not exclusively mediated in some way through the internet’* [2] remains relevant in a rapidly changing technological environment [3]. Its non-inferiority to ‘traditional’ educational methods has been largely accepted for many years in both healthcare [4] and wider educational domains [5]. Elearning’s potential for flexibility, scalability, and engaging learners offers a myriad of opportunities for educators [6, 7]. Elearning has perhaps been viewed as a panacea to some of the challenges (such as increasing student numbers and financial costs) of educating the next generation of healthcare professionals, rather than a learning enhancement opportunity.

Opportunities have inherent risk. Research has established that transposing from one medium (traditional taught courses) to a ‘trendy new one’ (a variety of elearning platforms) may be pedagogically deficient [8]. Existing literature also describes broad categories of obstacles to engaging with elearning, largely relating to practical and technical difficulties around access, quality concerns and a lack of support [9–11]. Learner experience, however, remains relatively unexplored.

Pragmatic acceptance of the fact that elearning is ‘part of the current educational landscape’ should direct research efforts to focus on ‘when’ and ‘how’ to use it most effectively [12, 13]. In other words, clarification research is required [6, 14, 15]. Our study addresses this clarification agenda, aiming to shed light on why elearning might be challenging at the level of the learner. We identified a need for a rigorously conducted exploratory study to probe this further. The research question was to explore students’ perceived obstacles to engaging with elearning.

## Methods

### Study design

We chose a qualitative approach, given the exploratory nature of our research question. We used a rigorous form of thematic analysis informed by principles of grounded theory [16]. This inductive approach generated a rich understanding, firmly grounded in participant experience. Ethical approval for this study was obtained from the Queen’s University Belfast (QUB) School Joint Research Ethics Committee. The Consolidated Criteria for Reporting Qualitative Research (COREQ) provide a focus for describing our approach and reporting our findings [17].

### Research setting

**Fig. 1:**
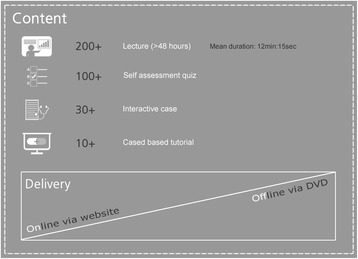
The elearning resource

The research was conducted with medical students at Queen’s University Belfast (QUB); a major academic institution in the United Kingdom (UK). Elearning is increasingly used throughout the 5 year course with students accessing through a sophisticated portal. It is particularly embedded in the third year, where from 2007 it has replaced all year-group wide traditional lectures. This third year elearning resource was intended to complement clinical experience within geographically dispersed hospital placements. Anticipated added advantages of blending learning in this way included ‘just in time learning’, the flexibility to link theory with clinical practice, and to aid revision. The elearning is also provided on DVD (several students refer to it as “the DVD”) to circumvent temperamental internet access in some peripheral hospitals (Fig. 1).

### Participants

Fourth year students were chosen as a focus for this study due to their recent experiences of a substantial elearning resource. Recruitment took place face-to-face during campus based teaching sessions and was done by HR. Participants were asked to devote up to one hour of a scheduled lunch break to take part in a focus group session. Lunch was provided but participation was not otherwise incentivised. Selection was purposeful; aiming for a mix of male and female, school-leaver and graduate entry students. There were no drop outs between recruitment and completion of the focus group sessions. A written information sheet provided to all participants set out HR’s motivations for conducting the study as part of a postgraduate degree. All participants provided full written consent before data collection (Table 1).

**Table 1:** Participant characteristics

Focus Group	Number of Participants	Male	Female	Age Range	Graduate Entry Students	International Students
1	10	6	4	21–23	0	0
2	11	3	8	21–25	2	0
3	10	6	4	21–22	0	0
Overall	31	15	16	21–25	2	0

### Data collection

KM and CT are faculty staff, who had previously conducted digital evaluations regarding student elearning experiences. Familiarisation with these responses provided sensitising concepts for development of an initial topic guide. This was piloted to ensure ease of understanding and designed to allow participants to follow a chronological narrative of their experiences.

HR conducted the focus groups. At the time of data collection she was a postgraduate researcher with no prior relationship with any participant. She had completed an intensive 2 day course on qualitative interviewing techniques prior to study commencement. Although a qualified clinician, participants did not know her in this context nor would they associate her with ‘university authorities’.

We selected focus groups as the data collection tool rather than individual interviews with the aim of explicitly utilising group interaction as part of the method. Focus group interviews were semi-structured. In keeping with principles of grounded theory, the initial topic guide was developed iteratively over the course of data collection, guided by previous participant responses. An example topic guide is available (Additional file 1). By encouraging participants to question each other and exchange anecdotes and experiences, we explored the full range of participant perspectives, and were able to probe more deeply to challenge their expressed views [18]. Focus groups took place in a private but informal space on campus. Sessions ranged in duration from 28 to 38 mn and an observer was present during two of the sessions. Both interviewer and observer made some brief field notes. These, together with HR’s fieldwork notebook enabled reflection on practicalities of conducting the focus groups and provided notes on interactions which were considered in conjunction with the relevant transcripts. Focus groups were audio recorded, anonymised and transcribed by HR.

Recruitment cycles took place at 6 week intervals, with parallel data transcription and analysis as advocated by Corbin and Strauss [16]. This allowed recruitment to continue until data saturation was reached; by this we mean that no new concepts or themes pertaining to perceived obstacles to elearning engagement were being identified in the data. Two members of the research team (HR and KM) independently assessed and agreed on saturation point.

### Analysis

Our analysis aimed to generate key themes firmly grounded in participant experience. Analysis was an inductive process, with no ‘a priori’ theoretical assumptions. Analysis was an immersive process with early analytical thoughts (collated as written ‘memos’) developed from the point of data collection. HR and KM independently and inductively coded the transcript of the first focus group. We refer to a ‘code’ as a label for a section of data relating to a particular idea. NVivo software (version 9 QSR International (UK) Limited) facilitated storage, retrieval and organisation of coded data. HR and KM met after the independent coding of the initial transcript to discuss and agree on a ‘codebook’ to facilitate further analysis. Coding and grouping of codes into categories was an iterative process and further codes were added as data collection progressed. Development of categories into themes was an evolving and non-linear process. Theme derivation, though inevitably influenced by the published literature, always aimed to be grounded in the data. No particular theoretical assumptions sensitised the approach to these data. Data coded within one category was explored and similarities and contradictions searched for among other categories. The process continued until the research team felt that the themes encapsulated the breadth and depth of participant experience. A participant checking exercise was carried out with consenting participants provided with a summary of themes and feedback being invited.

## Results

**Fig. 2:**
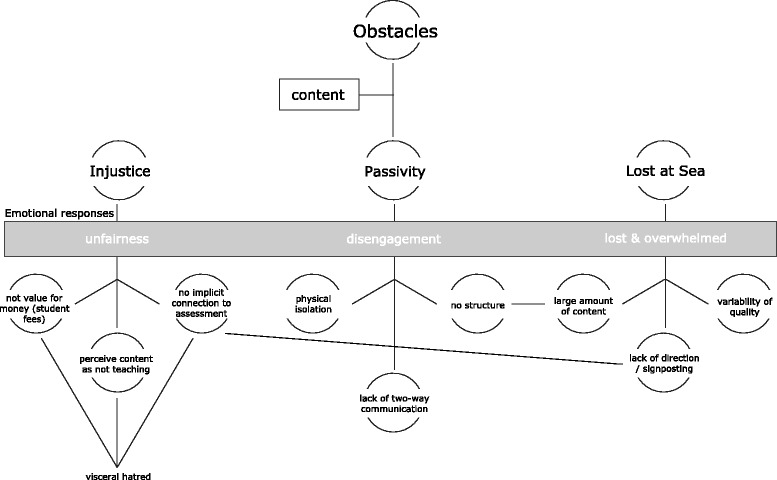
Concept map of obstacle themes

Participants described many perceived obstacles to their engagement with available elearning. Three key themes emerged from analysis. *Injustice* is a powerful theme where students conveyed a sense of resentment: the idea that they were somehow being ‘done out of’ the education that they deserved. *Passivity* draws on the sense that the participants felt a lack of control – that they were ‘passive recipients’ of elearning material. *Lost at sea* relates to unfamiliarity with this approach to learning and a sense of being overwhelmed. Issues relating to the actual content of the elearning underpin these themes which cannot be understood in isolation (Fig. 2).

### Injustice

This theme encapsulates unfairness. Students understood education as being a face-to face transaction between lecturer and student, delivered by traditional live teaching methods such as lectures and tutorials. They considered the digital elearning elements to be of intrinsically lower value than face-to-face teaching. Indeed, there was a cultural perception, reinforced by senior clinical staff, that elearning was not ‘real’ teaching at all.

‘Quite daunting wasn’t it? You were just given this massive eh, load of lectures and told, “right, you’re not going to be taught on it, but you’re going to be examined on it, so off you go.”’ (FG1M1)

‘Our consultant used to call it the £3500 *[annual tuition fee]* DVD’ (FG3M5)

Participants perceived that elearning ‘did them out of’ the education they felt entitled to, given that they pay substantial tuition fees. Participants felt that the elearning resource in some way represented medical education ‘on the cheap.’ In that sense, it violated the educational agreement that they felt they had signed up to with the institution, with students left feeling isolated and unsupported. Ensuing resentment created an obstacle to their engagement with elearning.

‘I found things very very frustrating, basically like you went into third year and an introductory week of lectures, and they hand you this DVD, give you these placements, and are like, “there you go, go learn what you need for the year.” And then we’ll see you in June and examine you.’ (FG1M3)

The strongest expressions of injustice were in relation to assessment. Learning was strongly driven by the prospect of end-of-year assessments. Given the weight of content within the medical curriculum, students strategised according to what was likely to come up on examinations. Consequently, across all focus group sessions, and echoed by most participants, was a sense that the elearning resource wasn’t directly contributing to assessment processes.

‘It’s just I don’t, I mean at the end of the day, when I did my exam, I realised that it was a waste of time, and it annoyed me.’ (FG1F3)

A compounded sense of frustration, resentment and visceral anger as the result of these interacting influences came across:

‘I grew to hate it more and more as the year went on, when you realised how vast it was and the things you just didn’t think you needed to know.’ (FG3F4)

‘Sometimes you just wanted to you know, snap [the DVD] in two.’ (FG3F3)

### Passivity

This encompasses a lack of compulsion to engage with the material, given the lack of a formal framework or timetable to which they were accountable, and a sense that the participants felt somehow passive recipients of the material.

‘The fact that it’s not compulsory for you to go home and do it every night like, you can just leave it and then let it all gather up.’ (FG1M3)

‘Yeah, if you don’t have like lectures and stuff to go to it is very easy to stow away and forget about it.’ (FG2F7)

This appears inextricably linked with the idea (described further within lost at sea theme) that the volume of the elearning was overwhelming. Failing to ‘get stuck in’ early to the resource was only going to make the situation more challenging.

‘I think just the fact that you can just leave it for ages, and then all that people think you have to do is just watch like ten lectures per night, but I think…no, but like you can’t do that.’ (FG2F2)

Participants viewed the elearning as something to be ‘got through’. Although they were apparently engaging with the material, some were not doing so in an active manner: they were behaving as passive recipients, with any learning somewhat incidental. Although they could ‘tick off’ that they had ‘completed’ a particular section of the elearning, ‘completion’ of these sections didn’t mean that they were engaged in any active learning process.

‘Like it was just sort of there in the background, so I could tick it off, but…’ (FG2F4)

They felt they had been detached and disengaged from their previous learning experience within conventional structures, leaving them feeling untethered and lacking in agency. The medical students were previously accustomed to attending mass lectures with their attendant social interactions with peers and faculty. They were now working alone at a computer, and found this physical isolation challenging.

‘I mean, there is no contact with anyone during the year.’ (FG1M2)

‘It’s quite a solitary thing as well.’ (FG1F3)

The passivity engendered by being forced to learn in this medium brought about a sense of disenfranchisement. This manifested as a level of disengagement which at times veered into passive resistance.

‘I’d be on the internet for other things, so it might just be up there in a different window.’ (FG2F 5)

‘A lot of the time you’re just sitting staring at the screen thinking when is this going to end *(laughter)* looking at the timer and going… *(laughter)*’ (FG1M2)

Thus passivity goes beyond a lack of compulsion to engage with the material and beyond distractions. There is a sense that the elearning was somehow ‘washing over’ participants. They were aware of and were exposing themselves to the content, but they did not feel actively engaged, constructing themselves as passive recipients. Furthermore, their resentment towards the material at times resulted in active disengagement.

### Lost at Sea

This theme encompasses feelings of being lost and overwhelmed with how to approach the elearning. The volume of material in the elearning resource was itself perceived as an obstacle. Participants described being overwhelmed and daunted by the load.

‘You were constantly thinking, “I have so much left to do.”’ (FG1M1)

‘It was overwhelming when you had the checklist and you’re looking at all the lectures and you’re like, “oh my goodness, how am I supposed to get through all these?”’ (FG3F3)

Not only was the volume of material an obstacle, but a perceived lack of direction as to how to approach the resource hindered participants. This could be described as a lack of signposting within the elearning resource. Taking the resource as a whole, participants felt that they didn’t know on which aspects to focus their learning. They felt lost as to how best to allocate their time.

‘There was no kind of guidelines as to you should have this much done by such and such a time, or this much done by this but I suppose it’s hard to do that, with people doing different things and different circumstances.’ (FG2M1)

References to assessment were again pervasive. Participants perceived the lack of signposting a particular obstacle as it related to their assessment processes: they wanted to know what they needed to know in order to pass their exams.

‘Telling us maybe that, that not everything is examinable, that, you know there are specific parts, and to point us to them is probably what would have been to the best advantage.’ (FG1M1)

Variability in length, format and quality (of production and content) of different components of the elearning resource was interpreted as an obstacle by some participants. This contributed further to a feeling of being lost in that they found structuring their learning and allocating study time challenging.

## Discussion

This investigation was part of a larger programme of work, and it should be recognised that there are many different perspectives on elearning. While this study was about identifying problematic issues, we are aware that many students engage positively with elearning.

In recent years students have become accustomed to high fidelity images and production quality through the use of sophisticated gaming and social media platforms. It would be unrealistic for medical schools to attempt to compete in terms of production quality. Indeed, we know broadly that students will accept ‘good enough’ quality. Inaudible sections (such as the speaker ‘drifting off into nothingness’ as related by one of our participants) will, however, inevitably evoke student discontent. A balance needs to be struck with technical and production quality of a realistic but acceptable standard. There are many practical measures that teams can take in terms of considering aesthetic appeal of learning resources [19]. Educators must, however, compete with other resources in terms of quality of content. This must be factually correct and reflect relevant recent developments in order to maintain credibility with students.

We know that today’s students are immersed in a technologically rich environment [20]. Potential distractors are ubiquitous and ‘multitasking’ may well affect learning [21]. There is a need, however, to accept and even embrace such environmental and cultural influences [19]. It is a task for educators to work with this challenge, rather than against it. Student frustration with elearning is multifactorial and may be brought about by a combination of difficulties with learning content and responses to potential distractors. It does, however, necessitate acknowledgement that it constitutes an emotional response to a learning activity.

Published studies of elearning interventions describe obstacles in terms of content of the resources, social isolation and unfamiliarity with the approach [9–11]. In our study, students described strong emotional responses to elearning material. ‘Injustice’ and ‘passivity’ are powerfully emotive words. Our explorative study inductively found that students experienced these strong emotions during their engagement with elearning material, and that in consequence their learning may have been impeded.

In recent years, there has been a resurgence of interest in the role of emotion across the breadth of educational scholarship and specifically within the health sphere [22]. Academic performance is no longer solely conceptualised as a cognitive activity, relatively free from emotional and motivational considerations. Emotion is not easy to define. Artino has written extensively about emotion and its relevance to healthcare professions education [23, 24]. He defines emotion as, ‘*an acute, intense, and typically brief psycho-physiological change that results from a response to a meaningful situation in an individual’s environment.’*

Emotions influence cognitive resources [22, 25]. In considering why this might be, the concept of *achievement emotions* becomes important. In 2006 Pekrun describes these as, ‘e*motions tied directly to achievement activities or achievement outcomes’* [26]. Achievement activities encompass many tasks encountered by students in the health professions; working to understand a clinical problem, participating in a ward round, or listening to a lecture, which could be in traditional or elearning format. Thus the injustice (encapsulated by the study participants using terms such as ‘hate’ and ‘unfair’) and passivity (‘bored’, ‘frustrated’) directed towards the activity of engaging with an elearning resource are examples of negative achievement emotions. In general, positive achievement emotions, together with associated appraisals of control and value, are thought to exert adaptive effects on learning, such as use of deep processing strategies actively linking new information to existing knowledge [26]. Negative achievement emotions would exert non-adaptive effects such as reverting to the use of superficial processing strategies, for example rote learning of facts [27].

Participants did indeed describe a checklist driven, rote learning approach to the material. Despite ‘great intentions’ of integrating their elearning ‘theory’ with real clinical encounters, there was evidence that they were not actually utilising such deep learning strategies. Students experienced negative emotional responses to the elearning, which was characterised as a low value resource over which they had little control. Negative emotional responses to educational technology in this setting caused students to adopt maladaptive learning strategies.

Control and value concepts of Pekrun’s achievement emotions framework [26] offer potentially useful insights that could aid educators. The intrinsic value placed by students on elearning resources could be increased by emphasising relevance to future professional practice. Perceptions of extrinsic worth could be challenged by making explicit links to assessment. Control appraisals relate largely to competence perceptions such as how confident an individual feels about a specific task [27]. Where the students in our study were describing being ‘lost at sea’ in a large and poorly signposted resource, this could be interpreted as low level of individual control. Clearer signposting alongside appropriate and focussed training to develop students’ self-confidence with both elearning technology and a more self-directed approach to learning could ultimately influence academic outcomes in the broadest sense. The fact that students (especially school leavers) are ill prepared for independent learning is widely recognised [28, 29].

### Strengths of this study

This study was developed to address a gap in the existing published literature. To the authors’ knowledge, this is the first study designed with the specific focused aim of exploring student obstacles to elearning in a health professions education setting. Results were developed through a highly inductive and reflexive process.

### Limitations of this study

This research was contextually situated within one UK institution. Qualitative exploration, though not necessarily generalisable, was felt to be appropriate to our research question. By providing an account of the context of this elearning resource, we believe that findings are relevant and theoretically transferrable to others working in similar contexts within health professions education.

### Directions of future research

This study explores student perceived obstacles to elearning. There are other stakeholders involved, from those more focused on the content such as educators, and those more focused on delivery such as developers. Future research could consider exploring their perspectives on obstacles to elearning engagement. Another consideration is that focusing on elearning in isolation is of limited value in today’s technological era. Research focusing on students as independent adult learners, rather than on the technology (which is here to stay and will develop faster than research into it) could be of more practical benefit.

## Conclusions

Emotional obstacles described by students in response to elearning offer new insights into engagement challenges in environments rich in digital distractors. Injustice and passivity are negative achievement emotions experienced by students engaging with elearning, which mitigate against intended learning outcomes of the material. This may be a function of the proportion of curricular content represented electronically. Appreciation of the motivating and demotivating factors at play in how students interact with elearning will be of use to curriculum developers, educational technologists and any individuals seeking to enrich their educational offering with technology enhanced delivery.

## Abbreviations

DVD, digital versatile disk or digital video disk; FG(x)M/F(x), focus group (number) Male/female participant (number); HR/KM/CT, Helen Reid/Kieran McGlade/Clare Thomson; QUB, Queen’s University, Belfast; UK, United Kingdom

## Additional file

Additional file 1: Sample topic guide (semi-structured for focus groups). (DOCX 13 kb)
