# Supplementary material for: Content and discontent: a qualitative exploration of obstacles to elearning engagement in medical students
Source: BMC Med Educ. 2016 Jul 22;16:188. doi: 10.1186/s12909-016-0710-5 (PMC4957903; doi:10.1186/s12909-016-0710-5)
Supplement: Additional file 1: — Sample topic guide (semi-structured for focus groups). (DOCX 13 kb) [file 12909_2016_710_MOESM1_ESM.docx]

Sample topic guide (semi-structured for focus groups)

Can you describe what your thoughts were on starting out with the DVD/website material at the start of third year?

How did you approach covering the material available?

Did any of your friends/colleagues go about this in a different way?

Can you explain a bit more about that?

Did the way you approached the material change at any point through third year?

Overall, how would you describe your experiences with the 3rd year elearning?

What barriers or difficulties did you encounter with the elearning?

Can you think of anything which might have helped you to get more out of the elearning material?

Can you think of anything which might have helped you use the material more efficiently?
